# Supplementary material for: Pre-Clinical Remote Undergraduate Medical Education During the COVID-19 Pandemic: A Survey Study
Source: Res Sq. 2020 Jun 10:rs.3.rs-33870. Preprint. [Version 1] doi: 10.21203/rs.3.rs-33870/v1 (PMC7336697; doi:10.21203/rs.3.rs-33870/v1)
Supplement: 1 [file Appendix.pdf]

Appendix 1. Qualtrics survey instrument.

Pre-Clinical Remote Learning Quality Improvement

Start of Block: Default Question Block

Q1 What year are you currently enrolled in at the school of medicine?

○ MS1 (1)

○ MS2 (2)

Q2 How has the transition to remote learning affected the **quality of instruction** you receive for each of the following curricular components?

	Very negatively affected (1)	Somewhat negatively affected (2)	Neutral (3)	Somewhat positively affected (4)	Very positively affected (5)	N/A: I did not take this course via remote learning (7)
Lecture-based learning (1)	○	○	○	○	○	○
PBL (2)	○	○	○	○	○	○
POM (3)	○	○	○	○	○	○
Anatomy (4)	○	○	○	○	○	○
Histology (6)	○	○	○	○	○	○
Ultrasound (7)	○	○	○	○	○	○
ACA Preceptorship (8)	○	○	○	○	○	○
Pre-clinical electives (9)	○	○	○	○	○	○

Q3 How has the transition to remote learning affected **your participation** in each of the following curricular components?

	Very negatively affected (1)	Somewhat negatively affected (2)	Neutral (3)	Somewhat positively affected (4)	Very positively affected (5)	N/A: I did not take this course via remote learning (6)
Lecture-based learning (1)	○	○	○	○	○	○
PBL (2)	○	○	○	○	○	○
POM (3)	○	○	○	○	○	○
Anatomy (4)	○	○	○	○	○	○
Histology (5)	○	○	○	○	○	○
Ultrasound (6)	○	○	○	○	○	○
ACA Preceptorship (7)	○	○	○	○	○	○
Pre-clinical electives (8)	○	○	○	○	○	○

Q4 I find the following resources valuable for my remote learning.

	Strongly Disagree (1)	Disagree (2)	Neutral (3)	Agree (4)	Strongly Agree (5)	N/A: I have not used this resource (6)
Online question bank subscription (1)	○	○	○	○	○	○
Online textbooks (2)	○	○	○	○	○	○
Podcasted lectures (4)	○	○	○	○	○	○
Online office hours/review sessions (5)	○	○	○	○	○	○
Zoom Pro Account (6)	○	○	○	○	○	○
Complete Anatomy (7)	○	○	○	○	○	○
JOVE Science Education (8)	○	○	○	○	○	○
Aquifer (9)	○	○	○	○	○	○
OnlineMed Ed (10)	○	○	○	○	○	○
iPad/Tablet (11)	○	○	○	○	○	○
Laptop (12)	○	○	○	○	○	○
Other: (13)	○	○	○	○	○	○

Q5 Since implementation of remote learning, how much additional money have you paid out of pocket for educational resources?

- $0-100 (5)
- $101-$500 (2)
- $501-1000 (3)
- $1000+ (4)

Q6 I felt adequately prepared to transition to remote learning.

- Strongly Disagree (1)
- Disagree (2)
- Neutral (3)
- Agree (4)
- Strongly Agree (5)

Q7 In terms of structure for the remote learning curriculum, which do you prefer?

- I prefer the flexibility of learning material at my own time and pace (1)
- I prefer having required modules and due dates (2)
- No preference (3)

Q8 Hypothetically, if some learning modules were required to be completed by certain due dates, how frequently would you prefer those due dates to occur?

○ Daily (1)

○ Every few days (2)

○ Weekly (3)

○ Biweekly (4)

○ Monthly (5)

Q9 I have found the following methods of communication effective in informing me about the changes in my education.

	Strongly Disagree (1)	Disagree (2)	Neutral (3)	Agree (4)	Strongly Agree (5)	N/A: I have not received communication via this method (6)
Emails (1)	○	○	○	○	○	○
Canvas (2)	○	○	○	○	○	○
Virtual Town Halls with SOM Deans (3)	○	○	○	○	○	○
Facebook Class Page (4)	○	○	○	○	○	○
University Website (5)	○	○	○	○	○	○
Other: (6)	○	○	○	○	○	○

Q10 Given the transition to remote learning, I feel that the examination process provides a fair assessment.

○ Strongly Disagree (1)

○ Disagree (2)

○ Neutral (3)

○ Agree (4)

○ Strongly Agree (5)

Q11 How do you feel your USMLE STEP 1 preparation has been affected by the transition to remote learning for your medical education curriculum?

○ Very negatively affected (1)

○ Somewhat negatively affected (2)

○ No change (3)

○ Somewhat positively affected (4)

○ Very positively affected (5)

Q12 Given the transition to remote learning, how prepared do you feel to begin clerkships?

○ Very unprepared (1)

○ Somewhat unprepared (2)

○ Neutral (3)

○ Somewhat prepared (4)

○ Very prepared (5)

Q13 Since implementation of remote learning, which of the following describes your residence location:

○ Stayed at UCSD graduate student housing (1)

○ Stayed in existing off-campus housing in San Diego (5)

○ Moved off-campus in San Diego (2)

○ Moved outside San Diego area (4)

Q14 Rate your agreement with the following statements:

	Strongly Disagree (1)	Disagree (2)	Neutral (3)	Agree (4)	Strongly Agree (5)
I have access to sufficient internet to meet the demands of remote learning. (2)	o	o	o	o	o
I have access to sufficient technology (ie a computer with a webcam, iPad, etc) to meet the demands of remote learning. (3)	o	o	o	o	o
Given my living arrangements, I have sufficient access to quiet study space to meet the demands of remote learning. (4)	o	o	o	o	o
Overall, my current living arrangements are conducive to remote learning. (6)	o	o	o	o	o

Q15 Rate your agreement with the following statements:

	Strongly Disagree (1)	Disagree (2)	Neutral (3)	Agree (4)	Strongly Agree (5)
Given the transition to remote learning, I still feel connected to UCSD School of Medicine. (1)	○	○	○	○	○
Given the transition to remote learning, I still feel connected to my classmates. (2)	○	○	○	○	○

Q16 If you have participated in any form of tele-health during this time, please comment on your experience:

____

Q17 What are the best components of the remote learning curriculum?

____

Q18 What gaps remain in the remote learning curriculum?

____

Q19 Which components of the remote learning curriculum should be continued in the standard curriculum in the future?

____

Q20 Finally and most importantly- please reflect on your own experience with remote learning for the pre-clinical curriculum. What would you like for course directors or others in medical education to better understand about the experience?

____

End of Block: Default Question Block
